# Total joint replacement may be a valuable treatment for Aboriginal and Torres Strait Islander people with osteoarthritis, but uptake is low

**DOI:** 10.1111/ans.17974

**Published:** 2022-08-13

**Authors:** Penny O'Brien, Sharmala Thuraisingam, Samantha Bunzli, Ivan Lin, Dawn Bessarab, Juli Coffin, Peter F. M. Choong, Michelle M. Dowsey

**Affiliations:** ^1^ The University of Melbourne, Department of Surgery St Vincent's Hospital Melbourne Melbourne Victoria Australia; ^2^ The University of Western Australia Western Australian Centre for Rural Health Geraldton Western Australia Australia; ^3^ The University of Western Australia Centre for Aboriginal Medical and Dental Health Perth Western Australia Australia; ^4^ Telethon Kids Institute Broome Western Australia Australia

**Keywords:** Aboriginal health, arthroplasty, health services, total joint replacement

## Abstract

**Background:**

Despite bearing a higher burden of osteoarthritis, little research has examined disparities in the access, utilisation and surgical outcomes associated with total joint replacement (TJR) among Aboriginal and Torres Strait Islander people.

**Methods:**

We conducted a retrospective cohort study to compare the sociodemographic and clinical characteristics of all Aboriginal and Torres Strait Islander and non‐Aboriginal patients who underwent primary hip and knee replacement at St Vincent's Hospital Melbourne between 1996 and 2019.

**Results:**

A total of 10 277 primary total knee or hip replacements were performed in the 1996–2019 study period, of which 49 (0.5%) patients identified as either Aboriginal and/or Torres Strait Islander. Aboriginal and Torres Strait Islander patients were younger (61.7 ± 11.8 vs. 68.3 ± 10.3 years; *P* < 0.001), recorded higher Body Mass Index scores (median (IQR), 36.0 (29.5–41.4) *vs.* 30.8 (27.0–35.3); *P* < 0.001) and were more likely to experience multiple co‐morbidities at the time of surgery. Despite these findings, Aboriginal and Torres Strait Islander patients did not experience higher complication rates and experienced comparable, clinically meaningful quality of life improvements 12‐months post‐surgery.

**Conclusions:**

TJR appears to be a valuable treatment option for Aboriginal and Torres Strait Islander people with end‐stage osteoarthritis. Our study was limited by the small number of procedures conducted in patients who identify as Aboriginal and Torres Strait Islander. Further research is needed to understand why uptake of TJR by Aboriginal and Torres Strait Islander people is low.

## Graphical abstract

Despite bearing a higher burden of osteoarthritis, little research has examined disparities in the access, utilisation and surgical outcomes associated with total joint replacement (TJR) in Aboriginal and Torres Strait Islander people. We conducted a retrospective cohort study to compare the sociodemographic and clinical characteristics of all Aboriginal and Torres Strait Islander and non‐Aboriginal patients who underwent primary hip and knee replacement at St Vincent's Hospital Melbourne between 1996 and 2019. A total of 10,277 primary total knee or hip replacements were performed in the 23‐year study period, of which 49 (0.5%) patients identified as either Aboriginal and/or Torres Strait Islander. Further investigation is needed to better understand the factors influencing low access and uptake of TJR in Aboriginal and Torres Strait Islander people.

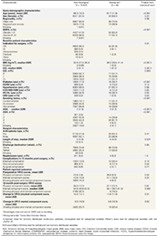

## Introduction

The Australian Orthopaedic Association National Joint Replacement Registry reported a total of 51 163 hip replacements and 66 729 knee replacements performed between 2018 and 2019 in Australia.^1^ These high‐demand, high‐volume procedures are considered safe and clinically effective interventions for people with end‐stage osteoarthritis.^2^ Whilst the burden of osteoarthritis is high globally, the burden of osteoarthritis disproportionately affects certain population groups within each country.^3^, ^4^, ^5^ In Australia, the prevalence and burden of osteoarthritis among Aboriginal and Torres Strait Islander people (respectfully Aboriginal people herewith) is higher, yet access to primary care, specialist services and total joint replacement (TJR) by Aboriginal people is low.^3^, ^6^, ^7^ Nationally, the relative risk of undergoing TJR is as low as 0.20 for Aboriginal women undergoing hip replacement, peaking at 0.49 for Aboriginal men undergoing knee replacement.^3^ Aboriginal people are also half as likely to be admitted for an elective surgical procedure and spend longer on surgical waiting lists when compared to non‐Aboriginal people.^8^, ^9^, ^10^, ^11^ Little research has examined disparities in the access, utilisation and surgical outcomes associated with TJR in Aboriginal people. Osteoarthritis is currently an unmet health need among Aboriginal people and calls have been made for timely, effective, culturally secure care to be implemented.^4^, ^5^ Further investigation is required to bolster our understanding of whether TJR is a safe and effective treatment which may be utilised to address the burden of osteoarthritis among Aboriginal people. No previous study has described the demographic and clinical characteristics of Aboriginal people undergoing TJR or compared preoperative and postoperative outcome data between Aboriginal and non‐Aboriginal patients. Therefore, the aim of this study was to describe and compare the sociodemographic and clinical characteristics of Aboriginal people and non‐Aboriginal people undergoing TJR.

## Methods

### Ethics

This project was approved by the St Vincent's Hospital Melbourne Human Research Ethics Committee (HREC185/19).

### Study design

We conducted a registry‐based retrospective cohort study at one of Australia's highest volume joint replacement services, a publicly funded metropolitan, tertiary hospital in Melbourne. St Vincent's Hospital Melbourne (SVHM) primarily services four metropolitan Melbourne municipalities, however, more than half of patients who seek care at SVHM live outside the primary catchment area and 11% travel from rural and regional Victoria. SVHM is the largest metropolitan provider of care to Victoria's Aboriginal community, with over 5000 occasions of care to Aboriginal adults annually. Based on hospital data from the Decision Support Unit, ~2.2% of acute hospital admissions at SVHM are patients who identify as Aboriginal, Aboriginal and Torres Strait Islander or Torres Strait Islander. Aboriginal people make up ~1.6% of the population in Victoria, excluding metropolitan Melbourne, and 0.5% of the population of Melbourne.^12^ Data were extracted from the St Vincent's Hospital Melbourne Arthroplasty Outcomes Registry (SMART),^13^ an institutional registry that prospectively collects clinical and patient‐reported outcome data in patients who have undergone elective hip and knee replacement at SVHM.  This registry commenced data capture in 1996 and from 1998 baseline demographic and clinical characteristics associated with surgery for all elective hip and knee procedures have been recorded. SMART contains preoperative and postoperative quality of life surveys, which were collected *ad hoc* until 2006 and then routinely in all patients from 2006 onward. SMART is also demographically representative of the national joint replacement population, and contains over 160 000 patient surveys, making this a unique resource for investigating patients who have undergone TJR.^14^

Registry records were extracted from SMART, allocated a study code and anonymised for all patients who underwent primary knee or hip replacement (TJR) between 1996 and 2019. Baseline demographic characteristics included age at time of surgery, sex, regionality and relative socioeconomic disadvantage measured by the Socio‐Economic Indexes for Areas rank (SEIFA; where deciles 1–5 represent areas that are more socioeconomically disadvantaged, and 6–10 represent areas that are more advantaged).^15^ Baseline patient characteristics included Indication for surgery (osteoarthritis, inflammatory arthritis, osteonecrosis, fracture, other), Body Mass Index (BMI), Charlson Comorbidity index (CCI), diagnoses of chronic conditions (type 2 diabetes mellitus, chronic obstructive airway disease, heart disease, chronic kidney disease). We also extracted clinical risk factors including hypertension, high cholesterol, active or past smoking status and American Society of Anesthesiologists illness grade (ASA). Surgical variables studied were arthroplasty type (hip/knee), length of stay, discharge destination (home/inpatient rehabilitation), complications (medical, surgical, wound) and unplanned readmission within 12‐months post‐surgery. Preoperative and 12‐month postoperative quality of life scores measured using the Veterans RAND 12‐item health survey (VR‐12), summarised in physical and mental component scores were also extracted.

### Statistical analysis

Data were analysed using Stata (version 16, StataCorp, Collage Stations, TX, USA). We used descriptive statistics to compare demographic and clinical characteristics of Aboriginal patients and non‐Aboriginal patients who underwent primary total knee or hip replacement between 1996 and 2019. We summarised normally distributed continuous variables using the mean and standard deviation and continuous variables with skewed distributions using the median and interquartile range. Categorical variables are presented as counts and percentages. We performed between‐group comparisons using unpaired *t*‐tests for normally distributed continuous variables, chi‐squared tests for categorical variables, Fisher's exact tests for categorical variables with cell count <5 and Wilcoxon‐Mann–Whitney tests for ordinal and non‐normally distributed variables, with significance level set at *P* < 0.05. Due to incomplete capture of ASA scores and quality of life surveys prior to 2006, we compared the demographic characteristics of those with complete and incomplete ASA scores and quality of life data.

## Results

### Socio‐demographics

A total of 10 277 primary knee or hip replacements were recorded in the 23‐year study period from 1996 to 2019. Socio‐demographic and baseline patient characteristics, surgical characteristics and patient reported outcomes are summarised in Table 1. As at December 2019, 49 (0.5%) patients identified as either Aboriginal, Torres Strait Islander or Aboriginal and Torres Strait Islander. Aboriginal patients in our study were on average younger than non‐Aboriginal patients, (61.7 ± 11.8 vs. 68.3 ± 10.3 years; *P* < 0.001) at the time of their surgery and there was little difference in the proportion of men and women between the two groups. There was a trend to suggest that Aboriginal patients were more likely than non‐Aboriginal patients to live outside major cities (26.5% vs. 17.0%; *P* = 0.08) and Aboriginal patients were more likely to reside in socially disadvantaged areas (65.3% vs. 41.5%; *P* < 0.001).

**Table 1:** Demographic and clinical characteristics of 10 722 patients who underwent total knee or total hip replacement between 1996 and 2019

Characteristic	Non‐Aboriginal *N* = 10 673	Aboriginal† *N* = 49	*P*‐value from statistical test‡
**Socio‐demographic characteristics**			
**Age (years), *mean* (*SD*)**	68.3 (10.3)	61.7 (11.8)	<0.001
**Sex (female), *n* (%)**	6511 (61.0)	34 (69.4)	0.23
**Regionality, *n* (%)**			0.08
*Major city*	8857 (83.0)	36 (73.5)	
*Regional and remote*	1815 (17.0)	13 (26.5)	
Missing	1 (0.01)	‐	
**SEIFA, *n* (%)**			<0.001
*Deciles 1–5*	4427 (41.5)	32 (65.3)	
*Deciles 6–10*	6239 (58.5)	17 (34.7)	
Missing	7 (0.07)	‐	
**Baseline patient characteristics**			
**Indication for surgery, *n* (%)**			0.31
*OA*	9630 (90.2)	45 (91.8)	
*IA*	589 (5.5)	3 (6.1)	
*Osteonecrosis*	336 (3.2)	‐	
*Fracture*	48 (0.5)	1 (2.0)	
*Other*	70 (0.7)	‐	
Missing	‐	‐	
**BMI (kg/m** ^**2**^ **), *median* (IQR)**	30.8 (27.0–35.3)	36.0 (29.5–41.4)	<0.001§
Missing	5 (0.05)	1 (2.0)	
**CCI, *median* (IQR)**	0 (0–1)	1 (0–2)	0.001§
**CCI, *n* (%)**			0.003
*0*	5946 (55.7)	17 (34.7)	
*1*	2809 (26.3)	15 (30.6)	
*2+*	1918 (18.0)	17 (34.7)	
**Diabetes (yes) *n* (%)**	1839 (17.2)	18 (36.7)	<0.001
**COAD (yes) *n* (%)**	569 (5.3)	5 (10.2)	0.13
**Hypertension (yes) *n* (%)**	6300 (59.0)	27 (55.1)	0.58
**CVD/IHD/AMI (yes) *n* (%)**	1204 (11.3)	6 (12.2)	0.83
**HC/HL (yes) *n* (%)**	3466 (32.5)	18 (36.7)	0.53
**CKD (yes) *n* (%)**	338 (3.2)	1 (2.0)	0.54
**Smoking status, *n* (%)**			<0.001
*Current smoker*	1080 (10.1)	17 (34.7)	
*Ex‐smoker*	2566 (24.0)	13 (26.5)	
*Non‐smoker*	7027 (65.8)	19 (38.8)	
**ASA, *− median* (*IQR*)**	2 (2–3)	3 (2–3)	<0.001§
**ASA, *n* (%)**			<0.001
*1*	301 (3.5)	‐	
*2*	4567 (52.8)	14 (29.8)	
*3*	3645 (42.2)	29 (61.7)	
*4*	133 (1.5)	4 (8.5)	
Missing	2027 (19.0)	2 (4.1)	
**Surgical characteristics**			
**Arthroplasty type, *n* (%)**			
Hip	5116 (47.9)	26 (53.1)	0.47
Knee	5557 (52.1)	23 (46.9)	
**Length of stay, *median* (*IQR*)**	5 (4–6)	5 (4–6)	0.53§
Missing	12 (0.1)	‐	
**Discharge destination (rehab), *n* (%)**			0.85
*Home*	7929 (74.6)	36 (73.5)	
*Rehab*	2695 (25.4)	13 (26.5)	
Missing	49 (0.5)	‐	
**Readmission (yes), *n* (%)**	871 (8.2)	4 (8.2)	1.0
**Complications in 12 months post‐surgery, *n* (%)**			
Medical complication	1334 (12.5)	10 (20.4)	0.10
Wound complication	968 (9.1)	6 (12.2)	0.44
Surgical complication	642 (6.0)	2 (4.1)	0.77
**Patient reported outcomes**			
**Preoperative VR12 scores, *mean* (*SD*)**			
Physical component scores	23.8 (7.8)	26.6 (9.3)	0.02
Mental component scores	43.8 (15.5)	41.1 (16.2)	0.24
Missing physical and mental component scores	1626 (15.2)	4 (8.2)	
**12‐month post‐surgery VR12 scores**			
Physical component score, ***mean* (*SD*)**	38.0 (12.2)	37.7 (13.1)	0.87
Mental component score, ***median* (*IQR*)**	54.8 (40.8–61.5)	50.1 (38.7–61.5)	0.39§
Missing physical and mental component scores	2166 (20.3)	8 (16.3)	
**Change in VR12 physical component score, *mean* (*SD*)**	14.2 (13.1)	11.6 (11.0)	0.21
**Change in VR12 mental component score, *mean* (*SD*)**	6.9 (16.5)	5.6 (18.3)	0.62
Missing physical and mental component scores	2420 (22.7)	10 (20.4)	

Percentages may not sum to 100% due to rounding.

† Aboriginal and/or Torres Strait Islander Status.

‡ Unpaired *t*‐test for normally distributed continuous variables, chi‐squared test for categorical variables Fisher's exact test for categorical variables with cell counts <5.

§ Wilcoxon‐Mann–Whitney test for ordinal and non‐normally distributed variables.

ASA, American Society of Anaesthesiologists illness grade; BMI, Body Mass Index; CCI, Charlson Comorbidity Index; CKD, chronic kidney disease; COAD, Chronic Obstructive Airway Disease; CVD/IHD/AMI, cardiovascular disease, ischemic heart disease, acute myocardial infarction; HC/HL, hypercholesterolemia/hyperlipidaemia; IA, inflammatory arthritis; OA, osteoarthritis; SEIFA, Socio‐Economic Index For Areas.

### Baseline patient characteristics

We noted no difference between Aboriginal and non‐Aboriginal patients' indication for surgery, with most patients (>90.0%) presenting with osteoarthritis. BMI was substantially higher in Aboriginal patients than in non‐Aboriginal patients (median (IQR), 36.0 (29.5–41.4) vs. 30.8 (27.0–35.3); *P* < 0.001). Aboriginal patients were more likely to experience multiple co‐morbidities and to have a diagnosis of type 2 diabetes (36.7% vs. 17.2%; *P* < 0.001). While there were no between group differences in the prevalence of any other individual co‐morbidities, Aboriginal patients recorded higher CCI scores, a reflection of multiple morbidity status. Aboriginal people were more likely to smoke, with just over one‐third being active smokers at the time of surgery (34.7% vs. 10.1%; *P* < 0.001). Aboriginal patients also had higher ASA scores at presentation for TJR (median (IQR), 3 (2, 3) vs. 2 (2, 3); *P* < 0.001).

### Surgical characteristics

No differences were observed when comparing the surgical characteristics of the two groups, including type of arthroplasty (hip/knee), length of hospital stay, discharge destination, rates of readmission or complications in the 12 months post‐surgery. We also observed no between group differences in surgical, medical or wound complications.

### Patient‐reported outcomes

There was little difference in both preoperative and 12‐month patient‐reported outcomes, except for preoperative VR‐12 physical component scores which were slightly higher among Aboriginal patients (mean (±*SD*), 26.6 (9.3) vs. 23.8 (7.2); *P* = 0.02). However, both groups showed comparable improvement 12‐months post‐surgery, recording similar change in both physical (mean (±*SD*), 11.6 (11.0) vs. 14.2 (13); 0.21) and mental (mean (±*SD*), 5.6 (18.3) vs. 6.9 (16.5); 0.62) component VR‐12 scores.

### Missing data

Missing data for ASA scores and VR‐12 surveys prior to 2006 was substantial. All other variables had <1% missing data (Table S1). For patients undergoing surgery between 2006 and 2019, over 95% had ASA scores and complete preoperative and follow‐up health surveys. We compared the demographic characteristics of those with complete and incomplete data (Table S1). The two groups were comparable except for those with complete data, who were more likely to have a diagnosis of high cholesterol.

## Discussion

There is a paucity of literature describing characteristics and health outcomes of Aboriginal people in the orthopaedic literature and to our knowledge, this study is the first of its kind. Overall, the proportion of people who identified as Aboriginal in our cohort was exceptionally small. In the 23‐year study period, 49 (0.5%) joint replacements were conducted in people who identified as Aboriginal, despite our hospital setting being considered a key provider of medical services to the local Aboriginal community. It has been reported that in some health services, patients are reluctant to disclose their Aboriginality for fear of receiving poorer care due to racial biases. While we cannot rule out that non‐disclosure of Aboriginal status was an issue in our study, SVHM was one of the first hospitals to establish a Koori Hospital Liaison Program in 1982 and feedback from the Aboriginal Health Unit indicates that this is unlikely.

A previous study exploring the variation in rates of TJR in Australia based on socio‐economic status, geographical location, birthplace and Indigenous status also reported low numbers of Aboriginal people undergoing TJR.^3^ After adjusting for age, Dixon *et al*. found that in the financial years 2005–2006 and 2006–2007 combined, Aboriginal men had approximately one‐third the number of hip replacements that would have been expected if they experienced the same procedure rates as non‐Aboriginal males of the same age, while this number was one fifth for Aboriginal women. Total knee replacements were also accessed at approximately half the expected rate based on incidence. This indicates that the uptake of TJR by Aboriginal people over the past 15 years is likely to have remained low. These barriers may be due, in part, to Aboriginal people more commonly utilising the public health system rather than services which require private health insurance.^16^ However, based on hospital data from the Decision Support Unit, only a small proportion (<3%) of patients undergoing TJR at SVHM are privately insured indicating this would have little impact on our cohort.

Low rates of TJR have also been recorded in Indigenous populations internationally, for example, First Nation Canadians access orthopaedic outpatient consultations, specialist services and TJR at substantially lower rates than non‐First Nations.^17^ Native Americans have the highest prevalence of arthritis of any population in the United States of America (USA),^18^ yet also utilise TJR at significantly lower rates than white Americans.^19^ In a study of 547 380 admissions for total knee replacement across eight racially diverse states in the USA, only 0.51% of admissions were for Native American patients. Racial disparities remained significant in adjusted analyses, implying that differences in patient demographics, health conditions and socioeconomic status only contributed in part to these racial disparities. Disparities in TJR also cannot be explained by the prevalence of osteoarthritis, which is higher among Indigenous populations globally.^5^ Instead, low rates of TJR are more likely due to disparities in healthcare access.^6^

Aboriginal people experience barriers to, and disparities in healthcare along the whole of the pathway to receiving surgical care, including access to appropriate primary and specialist services.^6^ In 2014–2015 Aboriginal people reported experiencing difficulty in accessing primary care services at 10 times the rate of the wider population,^20^ and Medicare claim rates for specialist services were 43% lower than for non‐Aboriginal people.^21^ Both primary and specialist services are essential steps in accessing appropriate referral pathways to surgical services and therefore, barriers to elective surgery may occur if these services are not accessible to those who need it. For those that do make it to the wait list for TJR at public hospitals, Aboriginal people wait longer for surgery than non‐Aboriginal people (median 278 days compared with 196 days for knee replacement; 188 days compared 120 for hip replacement).^10^

Furthermore, baseline demographic and clinical characteristics may have influenced the low rates of TJR by Aboriginal people in our study. Our study found that Aboriginal people undergoing TJR were significantly younger than non‐Aboriginal people. Younger patients may not be offered TJR due to concerns that the prosthesis may wear out and a revision may be required earlier than in older people undergoing TJR.^1^ However, the gap in life expectancy between Aboriginal and non‐Aboriginal people is estimated to be 8.6 years lower for Aboriginal males and 7.8 years for Aboriginal females.^22^ This may suggest that Aboriginal people experience a greater burden of osteoarthritis at an earlier age. Several factors may contribute to this, which was also reflected in our data. Modifiable risk factors such as obesity are known to contribute to degenerative joint conditions.^23^ Aboriginal people in our study experienced substantially higher BMI scores which may have also contributed to uptake of TJR at a younger age. Our cohort also experienced multiple comorbidities, type 2 diabetes and lifestyle risk factors such as smoking at higher rates, which is in keeping with current literature.^22^ Despite the complex chronic disease profile of Aboriginal people being well documented, the role of osteoarthritis and mobility limitations in managing chronic comorbidities is less recognised and warrants further exploration.^5^ With this demographic profile in mind, it may be worth clinicians and health services accepting a higher clinical risk profile when considering TJR as a treatment option for Aboriginal people. These findings are however contradictory to some evidence which suggests that a ‘treatment gap’ exists, whereby Aboriginal people are less likely to be offered surgical interventions as a treatment option even after adjusting for relevant patient and clinical characteristics.^24^ Further investigation is needed to understand whether low utilisation of TJR is a reflection of differences in healthcare access including socio‐economic circumstances and access to private health insurance, systematic or decision‐making biases and/or beliefs and knowledge about osteoarthritis and TJR among Aboriginal people, or a combination of these factors.

Despite their baseline characteristics, Aboriginal people in our study experienced comparable patient reported health outcomes to non‐Aboriginal people. We found no clinically important difference in the improvement of health‐related quality of life 12‐months post‐surgery and this was true for both mental and physical components of the VR‐12 health survey.^25^ Moreover, despite higher CCI and ASA rankings, the absence of difference in postoperative surgical or medical complications between Aboriginal and non‐Aboriginal people may suggest that programmes of preoperative medical optimisation are paramount in preparing candidates for TJR. This highlights the importance of routine peri‐operative care in ensuring good surgical outcomes in people with complex comorbidity profiles. Our results show that Aboriginal people with osteoarthritis benefited from undergoing TJR. The challenge now is to increase access to TJR within culturally secure healthcare environments so that more Aboriginal people who experience end‐stage osteoarthritis can benefit from this procedure.

## Strengths and limitations

Our main limitation was the exceptionally small number of procedures conducted in patients who identified as Aboriginal. This hindered our ability to perform more complex statistical analyses. At the outset of this study, we aimed to explore whether Aboriginal status was independently associated with postoperative quality of life outcomes for TJR. However, due to the small sample size we were unable to adjust our analyses for potential confounders. Furthermore, due to the small sample size, the Aboriginal cohort may not truly represent the Aboriginal population under study. We were unable to externally validate the cohort and therefore our study conclusions may have differed if our sample is not representative of the overall population. Our study should only be considered exploratory in nature and inferences made from our results are cause for further investigation rather than explanations. We recommend further work with additional data sets to examine possible relationships and confirm our findings.

Although we also encountered large amounts of missing quality of life surveys prior to 2006 in both cohorts, the characteristics of those with and without missing data were broadly comparable and therefore unlikely to have influenced our findings. Lastly, the patient‐reported outcomes in our study were measured using the VR‐12 survey, which has not been validated with Aboriginal people. Quality of life is dependent on cultural values and priorities. As such, this measure may not capture well‐being domains important to Aboriginal people such as respect, community, culture, spirituality and Country.^26^ The Good Spirit, Good Life tool is the first, validated quality of life tool which has been developed with and for older Aboriginal people.^26^ Incorporating Aboriginal values and worldview in the development of future quality of life measurement tools, for Aboriginal people with osteoarthritis should be a priority for further research.

## Conclusion

The burden of osteoarthritis disproportionately impacts Aboriginal people. TJR is an effective means of treatment for end‐stage osteoarthritis, which can significantly improve pain, function and quality of life. Our study also found that in the 23‐year study period, 0.5% of people undergoing TJR identified as Aboriginal, Torres Strait Islander or Aboriginal and Torres Strait Islander. Although Aboriginal people in our study undergoing TJR were younger, more likely to be overweight and more likely to have a diagnosis of multiple chronic diseases, they experienced comparable postoperative quality of life improvements 12‐moths post‐surgery to non‐Aboriginal people. This suggests that for Aboriginal people with end‐stage osteoarthritis, TJR may be a valuable treatment option. Our study was limited by the very small number of Aboriginal people undergoing TJR, and the reason for which remains unclear. However, the low numbers in this study indicate there may be disparities in healthcare access for this procedure. Further research is needed to understand the barriers to uptake of TJR by Aboriginal people.

## Conflict of interest

None declared.

## Disclosure statement

We are a group of Aboriginal and non‐Aboriginal clinicians and researchers with expertise in orthopaedic surgery, osteoarthritis and musculoskeletal health and cultural security. This publication was supported by a PhD scholarship offered through the Australian National Health and Medical Research Council Centre for Research Excellence in Total Joint Replacement (APP1116235). Sharmala Thuraisingam is supported by an Australian Government Research Training Program Scholarship. Professor Michelle M. Dowsey is supported by an Australian National Health and Medical Research Council Career Development Fellowship (APP1122526) and Professor Peter Choong is supported by an Australian National Health and Medical Research Council Practitioner Fellowship (APP1154203).

## Author Contributions

**Penny O'Brien:** Conceptualization; data curation; formal analysis; writing – original draft; writing – review and editing. **Sharmala Thuraisingam:** Data curation; formal analysis; writing – review and editing. **Samantha Bunzli:** Supervision; writing – review and editing. **Ivan Lin:** Supervision; writing – review and editing. **Dawn Bessarab:** Supervision; writing – review and editing. **Juli Coffin:** Supervision; writing – review and editing. **Peter F. M. Choong:** Supervision; writing – review and editing. **Michelle M. Dowsey:** Conceptualization; data curation; supervision; writing – review and editing.

## Supporting information


**Supplementary Table 1:** Demographic characteristics of complete and incomplete data
