# Supplementary material for: Total joint replacement may be a valuable treatment for Aboriginal and Torres Strait Islander people with osteoarthritis, but uptake is low
Source: ANZ J Surg. 2022 Aug 13;92(10):2676–82. doi: 10.1111/ans.17974 (PMC9804414; doi:10.1111/ans.17974)
Supplement: Supplementary file 1 — Supplementary Table 1: Demographic characteristics of complete and incomplete data [file ANS-92-2676-s001.docx]

| Supplementary Date Table: Demographic characteristics of complete and incomplete data | | |
| --- | --- | --- |
| **Characteristic (N=10,722)** | **Complete ASA and VR12 change scores**  **N=8,125** | **Incomplete ASA or VR12 change scores**  **N=2,597** |
| **SOCIO-DEMOGRAPHIC CHARACTERISTICS** | | |
| **Age [years], *mean (SD)*** | 68.3 (10.0) | 68.2 (11.0) |
| **Sex [female], *n (%)*** | 5,003 (61.6) | 1,542 (59.4) |
| **Regionality, *n (%)*** |  |  |
| *Major city* | 6,663 (82.0) | 2,230 (85.9) |
| *Regional & remote* | 1,462 (18.0) | 366 (14.1) |
| **SEIFA, *n (%)*** |  |  |
| *Deciles 1-5* | 3,308 (40.7) | 1,151 (44.4) |
| *Deciles 6-10* | 4,816 (59.3) | 1,440 (55.6) |
| **BASELINE PATIENT CHARACTERISTICS** | | |
| **Indication for surgery, *n (%)*** |  |  |
| *OA* | 7,449 (91.7) | 2,226 (85.7) |
| *RA/IA* | 422 (5.2) | 170 (6.6) |
| *Osteonecrosis* | 232 (2.9) | 104 (4.0) |
| *Fracture* | 6 (0.1) | 43 (1.7) |
| *Other* | 16 (0.2) | 54 (2.1) |
| **BMI [kg/m^2^], *median* [*IQR]*** | 31.2 [27.4-35.7] | 29.5 [25.7-33.9] |
| **Charlson Comorbidity Index (CCI) *– median [IQR]*** | 0 [0-1] | 0 [0-1] |
| **CCI, *n (%)*** |  |  |
| *0* | 4,562 (56.1) | 1,401 (53.9) |
| *1* | 2,084 (25.6) | 740 (28.5) |
| *2+* | 1,479 (18.2) | 456 (17.6) |
| **Diabetes (yes) *n (%)*** | 1,459 (18.0) | 398 (15.3) |
| **COAD (yes) n (%)** | 434 (5.3) | 140 (5.4) |
| **Hypertension (yes) *n (%)*** | 4,930 (60.7) | 1,397 (53.8) |
| **CVD/IHD/AMI (yes) *n (%)*** | 871 (10.7) | 339 (13.0) |
| **HC/HL (yes) *n (%)*** | 2,954 (36.4) | 530 (20.4) |
| **CKD (yes) *n (%)*** | 280 (3.4) | 59 (2.3) |
| **Smoking Status, *n (%)*** |  |  |
| *Current Smoker* | 813 (10.0) | 284 (10.9) |
| *Ex-smoker* | 2,214 (27.2) | 365 (14.0) |
| *Non-smoker* | 5,098 (62.7) | 1,948 (75.0) |
| **ASA, *n (%)*** |  |  |
| *1* | 291 (3.6) | 10 (1.8) |
| *2* | 4,316 (53.1) | 265 (46.6) |
| *3* | 3,411 (42.0) | 263 (46.3) |
| *4* | 107 (1.3) | 30 (5.3) |

Note: Percentages may not sum to 100% due to rounding
